# Venetoclax Combination Treatment of Acute Myeloid Leukemia in Adolescents and Young Adult Patients

**DOI:** 10.3390/jcm13072046

**Published:** 2024-04-01

**Authors:** Elena Chatzikalil, Kleoniki Roka, Panagiotis T. Diamantopoulos, Efthymia Rigatou, Georgia Avgerinou, Antonis Kattamis, Elena E. Solomou

**Affiliations:** 1 Division of Pediatric Hematology-Oncology, First Department of Pediatrics, National and Kapodistrian University of Athens Medical School, 11527 Athens, Greece; ehatzikali@gmail.com (E.C.); nikiroka@med.uoa.gr (K.R.); e_rigat@yahoo.com (E.R.); g.avgerinou@xahoo.gr (G.A.); ankatt@med.uoa.gr (A.K.); 2 “Aghia Sofia” Children’s Hospital ERN-PeadCan Center, 11527 Athens, Greece; 3 First Department of Internal Medicine, National and Kapodistrian University of Athens Medical School, 11527 Athens, Greece; pandiamantopoulos@gmail.com; 4 Department of Internal Medicine, University of Patras Medical School, 26500 Rion, Greece

**Keywords:** Bcl-2 inhibitor, venetoclax, acute myeloid leukemia, targeted therapy, adolescents, young adults

## Abstract

Over the past two decades, the prognosis in adolescents and young adults (AYAs) diagnosed with acute myeloid leukemia (AML) has significantly improved. The standard intensive cytotoxic treatment approach for AYAs with AML, consisting of induction chemotherapy with anthracycline/cytarabine combination followed by consolidation chemotherapy or stem cell transplantation, has lately been shifting toward novel targeted therapies, mostly in the fields of clinical trials. One of the most recent advances in treating AML is the combination of the B-cell lymphoma 2 (Bcl-2) inhibitor venetoclax with hypomethylating agents, which has been studied in elderly populations and was approved by the Food and Drug Administration (FDA) for patients over 75 years of age or patients excluded from intensive chemotherapy induction schemas due to comorbidities. Regarding the AYA population, venetoclax combination therapy could be a therapeutic option for patients with refractory/relapsed (R/R) AML, although data from real-world studies are currently limited. Venetoclax is frequently used by AYAs diagnosed with advanced hematologic malignancies, mainly acute lymphoblastic leukemia and myelodysplastic syndromes, as a salvage therapeutic option with considerable efficacy and safety. Herein, we aim to summarize the evidence obtained from clinical trials and observational studies on venetoclax use in AYAs with AML. Based on the available evidence, venetoclax is a safe and effective therapeutic option for R/R AML AYA patients. However, further research in larger cohorts is needed to confirm these data, establishing the benefits of a venetoclax-based regimen for this special population.

## 1. Introduction

Adolescents and young adults (AYA) represent a distinct age group, with ages ranging from 15 to 39 years ([1], https://www.siope.eu/encca/, assessed on 22 December 2023). Cancer epidemiology in AYAs differs from that in other age groups [1]. According to the ESMO/SIOP AYA working group, breast, thyroid, and testis cancers, followed by melanoma, are the most common neoplasms in AYAs [2]. There is also an increasing incidence of adult-onset cancers (colorectal, pancreas, and lung cancer) in the AYA population [2]. Regarding their therapeutic options, AYAs with a diagnosis of neoplasia face a disadvantage compared to pediatric patients and adults, presenting a high mortality with standard treatment strategies [3]. Specifically, AYAs present a poor survival compared to children for leukemias, lymphomas, central nervous system tumors, and some sarcoma subtypes (osteosarcoma and Ewing sarcoma), while they also present a poor survival compared to adults, but for different types of cancer, mainly for melanoma, breast, and colorectal cancers [4,5,6]. This evidence could partially be explained by their distinctiveness in biological features, despite the clinical and histopathological similarity of the cancer subtypes, compared to their pediatric and adult counterparts. This, however, favors the use of targeted therapeutic strategies, especially in types of neoplasms that currently remain intractable [7,8].

Acute myeloid leukemia (AML) is a molecular heterogeneous disease developed from the clonal expansion of hematopoietic precursor cells [9]. AML is characterized by a unique molecular age-associated signature, which affects the prognosis and survival in different age groups [10]. Pediatric AML patients mainly present balanced chromosomal rearrangements (translocations and inversions) and a lower frequency of somatic mutations (5–6 per pediatric sample) [11]. Frequent cytogenic rearrangements in pediatric AML patients are t (8;21) (q22;q22)/*RUNX1-RUNX1T1* and inv (16) (p13q22)/*CBFB-MYH11*, which are associated with a good prognosis, and 11q23/*KMT2A* (*MLL*), which is associated with an intermediate or adverse prognosis, depending on the *KMT2A* partner gene involved [11,12]. Alterations in the *RAS*, *KIT*, and *WT1* genes are more common compared to adults, and unbalanced abnormalities (e.g., chromosomes 5 or 7 monosomies) are rare and define a poor prognosis, while the presence of *FLT3-ITD*, *NPM1*, and *CEBPA* mutations is recommended to be identified in current therapeutic protocols [11,13,14]. AML cytogenic abnormalities in AYA patients present a similar pattern, characterized by a high frequency of balanced chromosomal rearrangements, except *NPM1* and *biCEBPA* mutations, which are associated with a normal karyotype and a favorable prognosis [15,16]. Frequent cytogenic rearrangements in AYAs are t (15;17) (q22;21)/*PML-RARA* (13%) and t (8;21) (q22;q22)/*RUNX1-RUNX1T1* (7%). Nearly 33% of AML mutations in AYAs concern the *NPM1* mutant phenotype, including *DNMT3A*, *IDH1/2*, and *FLT3-ITD* mutations, while “secondary-type” cytogenics, representing 13% of AML AYA patients, include *RUNX1*, *MLL-PTD*, and *ASXL1* mutations [16]. Mutations in *TP53* are of a lower frequency (8%) and are associated with a complex monosomal karyotype, resulting in a poor prognosis [17]. Regarding adult AML patients, a minority of them have balanced chromosomal rearrangements [11,18]. More common mutations in adult AML are *NPM1* and genomic subtypes characterized by mutated chromatin, RNA-splicing genes (e.g., *SRSF2*, *DNMT3A*, and *TET2*), and class-defining *IDH2* mutations [11]. *TP53* mutations present a higher frequency and *RUNX1* and *KMT2A* fusions present a lower frequency in adult AML compared to in other age groups, and both are associated with a poor prognosis, whereas complex karyotypes are considered quite common [11].

In all age groups, it is common for AML patients to suffer from relapse, months or years after treatment, whereas 5–10% of them will die due to disease complications or adverse effects of treatment [19]. Except differences between age groups in terms of cytogenic characteristics, it is worth noting, that, among the leukemias affecting AYAs, AML is of a high frequency (nearly 40%), statistically higher compared to that in pediatric (16%) and adult (31%) age groups [20]. AYAs diagnosed with AML present an overall cure rate ranging from 50% to 60%, with prognosis decreasing with increasing age, independently of other risk factors [21,22]. Interestingly, AYA leukemia survivors present higher mortality rates than the general population, which persists for decades after their diagnosis, despite the improvements in late mortality and long-term survival for AYA hematologic malignancies during the last decade [23]. This can partially be explained by the lack of therapeutic options for refractory or relapsed disease in this age group, which is usually excluded from novel therapeutic agents’ clinical trials, mostly because of the rarity of the neoplasms’ subtypes and due to ethical considerations as well [24]. Traditionally, AML in AYAs is treated with intensive chemotherapeutic regimens (anthracycline/cytarabine combination) and either consolidation chemotherapy or stem cell transplantation, presenting a low complete remission rate and dismal outcomes [25]. At this point, targeted therapy may enhance anti-leukemic efficacy and minimize treatment-related morbidity and mortality [26,27]. 

**Figure 1:**
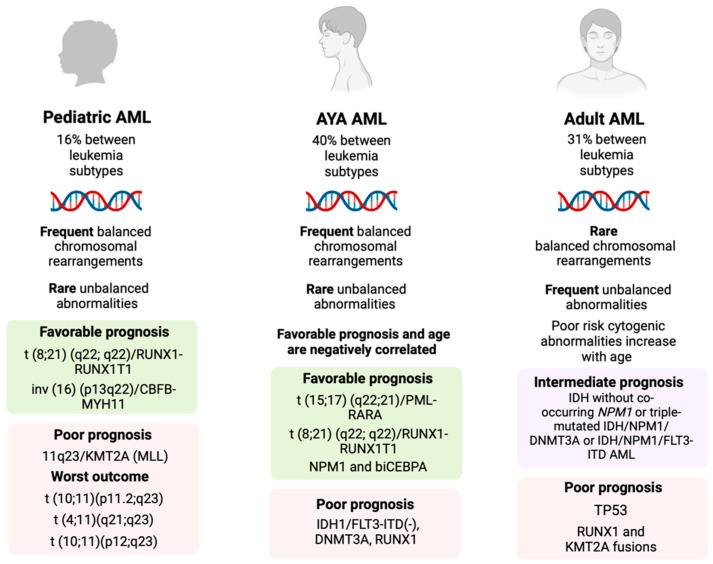
Schematic presentation of the distinct patterns of AML frequency and cytogenetic and molecular features between different age groups (image was created using Biorender software version 04, Licence #JC26MALGTX).

The aforementioned data regarding differences in AML frequency, prognosis, cytogenics, and molecular characteristics across the age spectrum (pediatric, AYA, and adult patients) are illustrated in Figure 1.

Venetoclax is an orally bioavailable specific B-cell lymphoma-2 (Bcl-2) inhibitor [28]. Bcl-2 is an important regulator of the apoptosis pathway by tightly controlling the release of cytochrome C from the mitochondria, the initiating step during apoptosis [29]. The overexpression of Bcl-2 is observed in AML cells, mostly those of the more immature FABn-M0/M1 subtypes [30]. In 2018, venetoclax was approved by the FDA for the treatment of adults with newly diagnosed AML who are aged 75 years or older, or who are ineligible for intensive induction chemotherapy [28,31]. Currently, venetoclax is used in combination with hypomethylating agents (azacitidine or decitabine) or low-dose cytarabine, and has been shown to be superior to hypomethylating agents alone [32]. The use of venetoclax in AYAs with AML has recently been reported in a small number of studies with a promising efficacy and safety for this age group [31]. However, the evidence establishing these reports has not been clearly defined and documented.

We aimed to review the currently available evidence for the use of venetoclax regimens in AYA AML populations. This was obtained from the medical literature, retrospective studies, and published or ongoing clinical trials. We performed a search in the PubMed and Medline databases with a combination of the following terms: “venetoclax”, “acute myeloid leukemia”, “adolescents”, “young adults”, “relapse”, and “refractory”.

## 2. Venetoclax Mechanism of Action and FDA Approval History

Apoptosis describes the orchestrated collapse of a cell, playing an important role in the development and maintenance of tissue homeostasis [33,34]. Cells undergo apoptosis through two different pathways, both of which result in the activation of the mediator of apoptosis, the caspase complex: the extrinsic pathway (death receptor pathway), activated by ligand-bound death receptors, mainly including TNF-TNFR1, FasL-Fas, and TRAIL-DR4/DR5, or the intrinsic pathway (the mitochondrial pathway), regulated by the members of the Bcl-2 family, which mediates and controls membrane permeabilization, a pivotal event in the mitochondrial pathway [34,35,36]. The Bcl-2 family consists of three groups of proteins, all of which contain at least one of four relatively conserved Bcl-2 homology (BH) domains, namely, the multidomain pro-apoptotic Bcl-2 group (Bak, Bax, and Bok), the multidomain antiapoptotic Bcl-2 group (Bcl-2, Bcl-XL, Bcl-w, A1, and Mcl-1), and the BH3-only domain pro-apoptotic group (BIM, PUMA, BID, BAD, NOXA, BIK, and BMF) [35,36].

The anti-apoptotic members of the Bcl-2 family (e.g., Bcl-2) usually present a high-level expression in human tissues [37]. The mitochondrial apoptotic pathway is normally controlled through the sequestering of pro-apoptotic members by the anti-apoptotic members of the Bcl-2 family [38]. In the case of neoplastic processes, these mechanisms are deregulated. Antiapoptotic proteins are increased, resulting in a reduced release of pro-apoptotic proteins and causing impaired membrane permeabilization, inhibiting apoptosis [39,40,41]. Oncogenesis is, therefore, associated with an abnormal expression of Bcl-2 family members, which makes tumor cells insensitive to apoptotic signals and gain growth advantages [39,40,41]. In view of this situation, the blockage of anti-apoptotic Bcl-2 family members could result in the restoration of the normal apoptosis of tumor cells.

Regarding hematologic malignancies, the anti-apoptotic molecules Bcl-2 and Mcl-1 are commonly highly expressed, playing a pivotal role in their biological characteristics via the dysregulation of their expression or by being associated with the cell-of-origin of the hematologic neoplasm [42]. Bcl-2 overexpression in all leukemias, especially in chronic lymphocytic leukemia (CLL) and AML, has been reported [43]. The pro-apoptotic BH3-only proteins interact with Bcl-2 via tight binding, inhibiting its anti-apoptotic function. By mimicking the action of the BH3-only proteins, a selective chemical inhibitor would induce mitochondrial apoptosis, compromising the normal mitochondrial pathway function [44]. Towards this direction, research has focused on small oligopeptide BH-3 mimetics targeting Bcl-2 (±BclxL) or Mcl-1 [42]. Navitoclax was the first Bcl-2 inhibitor used in clinical trials, with moderate results as monotherapy and severe dose-limiting toxicity [45,46,47]. Specifically, navitoclax co-targets BclxL, which plays a pivotal role in platelet survival, presenting an acute thrombocytopenic effect as a direct result of its use [48].

Venetoclax (ABT-199) has been specially designed to specifically target Bcl-2, presenting an increased affinity for the Bcl-2 protein (Ki < 1 nM) and a reduced affinity for BclxL (Ki > 100 nM) [29,47]. Venetoclax primarily acts by activating Bax proteins, resulting in mitochondrial outer membrane permeabilization, thus leading to cell apoptosis [49,50,51]. Apoptosis enhancement inhibits cellular growth, delaying tumor progression [49]. To our knowledge, venetoclax presents the aforementioned mechanism of action across the age spectrum, and, moreover, has advantages over traditional therapeutic drugs, partially due to the fact that Bcl-2 expression is non-essential in normal cells and is usually upregulated in malignancies [41]. This evidence combined favors investigating its use in AML, even in patients of younger ages.

**Figure 2:**
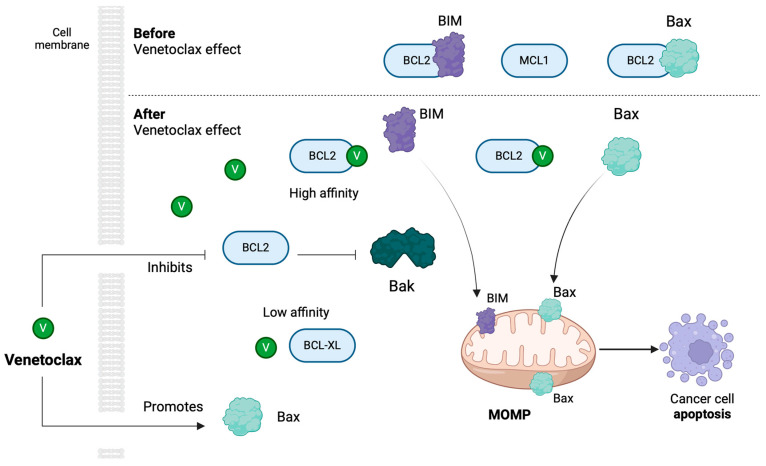
Schematic presentation of venetoclax mechanism of action. Venetoclax targets Bcl-2, presenting increased affinity for Bcl-2 and reduced affinity for BclxL, and activates BAX proteins, resulting in mitochondrial outer membrane permeabilization (MOMP), which leads to cell apoptosis (Image was created using Biorender software version 04, Licence #XP26MAAH3Z).

The venetoclax mechanism of action is illustrated in Figure 2.

Venetoclax was initially studied in clinical trials including patients with a diagnosis of CLL and non-Hodgkin lymphoma, presenting encouraging results in patients’ survival, especially for CLL, for which it was firstly approved by the FDA and EMA [44,52]. Specifically, in 2016, venetoclax was approved for the treatment of patients with CLL with 17p deletion who have received at least one prior therapy [53]. Two years later, venetoclax was approved for the treatment of patients with CLL or small lymphocytic leukemia (SLL) with or without a 17p deletion who have received at least one prior therapy (June 2018) [54,55]. Accelerated approval was granted in combination with azacitidine, decitabine, or low-dose cytarabine for newly diagnosed AML patients aged ≥75 years or with comorbidities that preclude the use of intensive chemotherapy (November 2018) [54,55]. In 2019, venetoclax was approved in combination with obinutuzumab for previously untreated patients with CLL or SLL, and in 2020, regular approval was granted, in combination with azacitidine, decitabine, or low-dose cytarabine, for newly diagnosed AML patients aged ≥75 years or with comorbidities that preclude the use of intensive chemotherapy [54,55].

Evidence shows that, as an AML therapeutic option, venetoclax has entered the fields of clinical trials more recently, considered as a very promising regimen in patients ineligible for intensive chemotherapy [56,57]. An up to date analysis of the use of venetoclax in different groups of AML patients, focusing on the AYA age group, is provided bellow.

## 3. Venetoclax in AML

AML is characterized by various cytogenic and molecular features, which develop a disease landscape complex to treat and create the need for multi-targeting chemotherapeutic regimens, being a challenge in drug development for nearly 50 years [56,57,58,59,60]. Currently, the standard therapeutic option for newly diagnosed AML consists of intensive induction chemotherapy with cytarabine and anthracycline, followed by consolidation therapy with cytarabine or allogeneic hematopoietic stem cell transplantation [61]. This results in a 60–80% complete remission (CR) with a 16–24 month median overall survival (OS) in younger patients and a 40–60% CR with a 9–12 month median OS in elderly patients [57,58,59,60,61]. With the available standard treatment options, more than 60% of AML patients across all age groups relapse [28]. Moreover, treatment-related toxicity underscores the need to develop new therapeutic regimens [62]. 

During the last ten years, a better understanding of the unique molecular signatures of different AML subtypes has led to the development of new therapies for patients with certain molecular characteristics [63]. *IDH* and *FLT3* inhibitors are now used in patients who present with these mutations [26]. These treatment options have improved patients’ survival, but lack durable efficacy and are not well-tolerated by all patients [64,65]. 

As mentioned before, Bcl-2 inhibitors have been used in various hematologic malignancies, mainly in combination with other chemotherapies, with promising results for patients’ survival, which led to their approval by the FDA and EMA for these types of neoplasms [28,46,47,66,67,68]. Their approval was based on the results obtained from the phase III VIALE-A and VIALE-C trials.

The VIALE-A trial was a multicenter randomized double-blinded placebo-controlled phase III study which included 431 patients with newly diagnosed AML, who were ineligible for intensive chemotherapy and were randomized in a 2:1 ratio to receive venetoclax (final dose: 400 mg/day) or a placebo combined with azacitidine [69]. Τhe patients who were treated with venetoclax/azacitidine presented a 34% reduction in risk of death compared to those who were treated with placebo/azacitidine, while the difference in CR was 66% vs. 28% for these patient groups, respectively [69]. The VIALE-C study was a randomized, double-blind, placebo-controlled, multicenter phase III study, which included 211 newly diagnosed AML patients ineligible for intensive chemotherapy randomized in a 2:1 ratio to receive venetoclax (final dose: 600 mg/day) or a placebo, combined with low-dose cytarabine (LDAC) [70]. Interestingly, patients who were treated with venetoclax/LDAC did not present statistical significance in the reduction in the risk of death (25%) compared to those who were treated with placebo/LDAC, while the difference in CR was statistically significant, specifically 48% vs. 13% for these patient groups, respectively [70]. Regarding venetoclax’s safety, thrombocytopenia and neutropenia were the most common adverse events reported in these studies, respectively [69,70]. 

Since its approval, venetoclax has been tested in combination with other chemotherapeutic agents, mainly with fludarabine, high-dose cytarabine, idarubicin, and liposomal daunorubicin [71,72,73]. Moreover, considering the rich mutational landscape of AML, venetoclax is currently under investigation combined with targeted options, specifically with *FLT-3* inhibitors (e.g., gilderitinib) and *IDH1* inhibitors (e.g., ivosidenib) [74,75]. There are many ongoing clinical trials evaluating these combinations’ safety and efficacy for AML patients. However, response rates, OS, chemoresistance, and reported adverse events vary between different patient cohorts [71,72,73,74,75,76,77]. 

It is worth mentioning that each patient’s mutational profile partially determines their sensitivity to venetoclax regimens. Specifically, *NPM1-*, *IDH1/2-*, *TET2-*, and relapsed or refractory *RUNX1*-mutated AML patients present a high sensitivity to venetoclax combination therapy [78,79,80,81,82,83], while patients carrying *FLT3*, *TP53*, *RAS*, or *PTPN11* mutations show a reduced sensitivity to venetoclax-based therapies. In particular, IDH1/2 mutations are present in 20% of AML patients, which makes venetoclax combination therapy a potential option for a large group of AML patients [84]. Moreover, patients diagnosed with a monocytic AML type, which is characterized by CD117 loss and the upregulation of CD11b, CD68, and CD64, show resistance to venetoclax combination therapy [85]. Furthermore, research has shown that Bcl-2 and other members of the mitochondrial pathway need to have a normal expression and function for venetoclax regimens to work properly. Decreased Bcl-2 levels and BAX inactivation are associated with a poor response to venetoclax regimens [86]. 

## 4. Venetoclax Combinations in the Treatment of AML in AYAs

AYAs traditionally present improved survival rates compared to elderly populations when diagnosed with AML [87,88,89,90,91]. On the other hand, it remains controversial whether their outcomes compared to those in pediatric populations = are inferior [14] or similar [92,93]. There are limited studies comparing the survival rates in children and AYAs to adults with AML [94]. However, the survival rates for adolescents and young adults with R/R AML certainly remain poor [25,95]. Disease-related factors, mainly the cytogenetic and mutational profiles, may affect patients’ survival, as well as patient-related factors, such as the socioeconomic patient profile [96]. Moreover, AYAs have less access to novel therapies due to their exclusion from clinical trials, especially compared to older adults, a fact that may also affect their survival [97]. 

Regarding novel therapeutic targets in the AYA age group, data are scarce [95]. Real-world evidence on venetoclax’s use as a therapeutic option for R/R AML in AYAs is limited [98]. Our literature search identified a few trials using venetoclax in AYAs (Table 1 and Table 2).

The VENAML study (NCT03194932) is a phase 1/2 dose escalation study from St Jude Children’s Research Hospital testing the dosage and efficacy of a venetoclax and cytarabine reinduction regimen in 38 pediatric patients with R/R AML (4 patients with primary refractory, 33 patients with relapsed AML, and 1 patient with relapsed mixed-phenotypic AML), in a pediatric (33 subjects aged 3–15 years), adolescent, and young adults’ cohort (6 subjects aged 15–19 years) [99]. During dose escalation, the participants received venetoclax orally once per day in continuous 28 day cycles at either 240 mg/m^2^ or 360 mg/m^2^, in combination with intravenous cytarabine every 12 h at either 100 mg/m^2^ for 20 doses or 1000 mg/m^2^ for 8 doses, with or without intravenous idarubicin (12 mg/m^2^) as a single dose [99]. The primary endpoint was the recommended phase 2 dose of venetoclax plus chemotherapy and the secondary endpoint was the proportion of patients treated at the recommended phase 2 dose who achieved CR or CR with incomplete hematological recovery [99]. The recommended phase 2 dose of venetoclax was found to be 360 mg/m^2^ (maximum 600 mg) combined with cytarabine (1000 mg/m^2^ per dose for eight doses), with or without idarubicin (12 mg/m^2^ as a single dose) [99]. From the AYA study population, one subject (18-year-old female), who carried the *FUS-ERG* mutation, was treated at first relapse without a prior hematopoietic stem cell transplant (HSCT), and achieved CR [99]. Three subjects, a 17-year-old male carrying *FLT3-ITD*, *MECOM*, *CBL*, *PTPN11*, and *WT1* mutations, a 15-year-old female presenting with dup (2) (q11.2q21) and *CEBPA* and *IKZF1* alterations, and a 16-year-old male carrying *PICALM-MLLT10*, *TP53*, *EXH2*, *PTPN11*, *NF1*, and *PHF6* mutations, were treated in the primary refractory setting and showed no response, partial response (PR), and PR respectively [99]. Finally, one subject (19-year-old male) with a *KMT2A* mutation and ins (10;11) (p13; q13q23) was treated in his second relapse, with a non-evaluable response, and one subject (17-year-old female) with R/R disease and *RUNX1-RUNX1T1* mutations achieved CR [99]. Response to treatment was determined by minimal residual disease (MRD) measured by flow cytometry in bone marrow samples [99]. Treatment-related toxicity was not analyzed individually for each patient [99]. It is worth mentioning that, for the total of 38 pediatric and AYAs participants, febrile neutropenia was reported in 63%, which is expected in heavily pre-treated AML populations, and grade 3 and 4 infections were reported in a frequency similar to that in other AML trials [99]. 

The SELCLAX study (NCT04898894) is a phase 1 expansion cohort study investigating the combination of Selinexor and venetoclax with chemotherapy (fludarabine, cytarabine ± granulocyte-colony stimulating factor) in pediatric and AYA patients with R/R AML/Acute Leukemia of Ambiguous Lineage (ALAL) [96]. Among the cohort’s AML patients, 11 were relapsed AML cases and 4 received venetoclax regimens for primary refractory AML [96]. As of 14 July 2023, 15 patients (aged 3–17 years, 2 adolescents ≥16 years of age) with R/R AML (n = 14) or ALAL (n = 1) were enrolled in the dose escalation phase [96]. In the dose level 1 (DL1) cohort, venetoclax was given at 360 mg/m^2^ per dose (max 600 mg) on days 1–21 in combination with selinexor at 40 mg/m^2^ on days 1, 8, and 15 [96]. For dose level 2 (DL2), venetoclax was dosed as in DL1, and selinexor was dosed at 40 mg/m^2^ twice weekly on days 1, 3, 8, 10, 15, and 17 [96]. From the AYA study population so far, one subject (17-year-old female), who carried *NUP98*, *NSD1*, and *FLT3-ITD* mutations, was treated with DL1 at relapse and another subject (16-year-old female), who carried *KMT2A* and *MLLT3* mutations, was treated with DL2. The first patient showed PR, but no response was seen in the second patient [96]. No treatment-related toxicity was reported in either of the two patients [96].

Currently, the Leukemia & Lymphoma Society (LLS) Pediatric Acute Leukemia (PedAL) are conducting a program of clinical trials evaluating the safety and efficacy of new agents in pediatric leukemia, setting a new standard of treatment for relapsed AML [100]. In Europe, the current project consists of a registry protocol (EuPAL2021 Registry) and a Master protocol with sub-trials (ITCC-101) [100]. The international ITCC-101/APAL2020D open-label phase III randomized multicenter sub-trial (NCT05183035) is the first sub-trial of the EuPAL2021 Registry evaluating the overall and event-free survival of children and adolescents with AML in second relapse without FLT3/ITD mutations [100]. Participants are randomized to fludarabine/cytarabine chemotherapy with filgrastim (FLAG) or FLAG combined with venetoclax, administered at 300 mg on Cycle 1 Day 1 and 600 mg on subsequent days of each 28 day cycle. A HSCT will be available after the first two cycles for patients who show a response [100]. The aim of this trial is to set a new standard of care for the second relapse of AML in children and adolescents, but to our knowledge, the results have not been reported yet [100]. The primary analysis will be performed approximately five years after the first patient is randomized [100].

Finally, the SAVE study (NCT05360160) will investigate the oral combination of the menin inhibitor SNDX-5613 (revumenib) combined with decitabine/cedazuridine (STX727) and venetoclax in AML, consisting of two parts [101]. The study includes patients with primary refractory AML and patients who relapsed with previous chemotherapeutic regimens [101]. The first part consists of investigating the maximum tolerable dose of SNDX-5613 that can be given in combination with decitabine/cedazuridine and venetoclax for patients with AML or those with a mixed-phenotype acute leukemia with a myeloid phenotype (MPAL), and the second part will evaluate the therapeutic potential of this regimen in AML/MPAL [101]. STX727 is given at 35 mg/100 mg daily on days 1–5, venetoclax at 400 mg daily on days 1–14, and revumenib at 113 mg every 12 h (dose level [DL] 0) or at 163 mg every 12 h (DL 1, used in phase II monotherapy) on days 1–28 with either posaconazole or voriconazole [101]. Maintenance with revumenib monotherapy is planned following HSCT for one year [101]. The early results of the study show that seven out of eight patients attained morphological remission, and MRD by flow cytometry was undetectable in three out of seven patients [101]. The early results show an acceptable safety and promising efficacy [101].

Regarding retrospective cohort studies, Winters et al. were the first to publish real-world evidence on pre-SCT venetoclax combination therapy in pediatric patients (n = 6, aged < 12 y.o.) and young adults (n = 2) who were not part of clinical trials [102]. The patients received venetoclax orally on a dose escalation (100 mg, 200 mg, and 400 mg on days 1, 2, and 3 respectively) and then 400 mg in 28 day cycles, combined with azcitidine at 75 mg/m^2^ for 7 consecutive days [102] Of the two young adult patients, the first (18-year-old female), who had FLT3-ITD, WT1, and NUP98-NSD1 mutations (“triple mutant” AML), achieved a morphologic leukemia-free state after being treated with a venetoclax/azacitidine combination plus gilteritinib [102]. The other patient (20-year-old male), who had FLT3-TKD, WT1, BCORL1, and GATA2 mutations and a karyotypic analysis with 46, XY, and t (2;14) (q22; q32) alterations, achieved CR after being treated with a venetoclax/azacitidine combination plus sorafenib [102]. In this study, the treatment-related toxicity of the venetoclax/azacitidine regimen was not analyzed for each patient, but for the total of eight participants [102]. The patients received SCT after post venetoclax/azacitidine treatment remission [102]. No grade 5 adverse events were reported, while the most common adverse event was neutropenia, which was reported for all eight patients, including the two young adults, and was well-tolerated with a median duration of 20 days [102].

These optimal results are contrary to those of another single-institution report of two cases, from which the one subject belonged to the AYA age group [90]. Specifically, the authors reported the case of a 26-year-old woman diagnosed with monocytic AML [t (6;11), mixed-lineage leukemia, (MLL) positive, *FLT3*-ITD negative] [98]. She was treated with conventional chemotherapy followed by an allogeneic HSCT and relapsed two years after induction [98]. She received azacitidine for two cycles without response, before she started combination treatment with azacitidine/venetoclax (titrated from 100 mg daily to a maximum of 400 mg daily) for three cycles [98]. However, the patient died of disease progression and a febrile syndrome before the first response evaluation [98].

Trabal et al. recently published the largest retrospective two-center cohort study on venetoclax regimens’ use in pediatric and AYA patients [103]. The study included patients aged ≤ 21 years with R/R AML, who received one or more cycles of venetoclax combined with other agents (hypomethylating agents, fludarabine, cytarabine, granulocyte-colony stimulating factor, and idarubicin—FLAG-IDA, cladribine, cytarabine, idarubicin, gemtuzumab, gilteritinib, sorafenib, or midostaurin, tyrosine kinase inhibitors, and arsenic trioxide) [103]. This study’s population consisted of 19 patients with relapsed AML who had received ≥ three prior lines of therapy, 16 patients who had received a prior bone marrow transplant, and 35 patients with unfavorable genetics, which will herein be described below [103]. Adolescents and young adults were dosed at the maximum FDA-approved dose of venetoclax of 400 mg [103]. Venetoclax dose reductions ranging from 30% to 75% were employed in patients with the concomitant use of CYP3A inhibitors. Among a total of 43 participants, 27 belonged to the AYA age group, with a median age of 20 years (range: 15–21) [103]. In total, 10 out of 27 AYA patients, with a median age of 20 years, achieved CR (cytogenics/molecular characteristics: *KMT2A*: 3 patients, *FLT3-ITD*: 1 patient, *WT1*: 3 patients, Monosomy 7: 2 patients, *NPM1*: 2 patients, *RAS*: 3 patients, *TP53*: 1 patient, *RUNX1-RUNX1T1*: 2 patients, Inv (3): 1 patient, and *IDH1/2*: 1 patient) [103]. A total of 1 out of 27 AYA patients (16-year-old female), carrying *KMT2A* and *STAT5* mutations, achieved PR [103]. Fourteen AYAs, with a median age of 18.5 years, achieved CR (cytogenics/molecular characteristics: *KMT2A*: one patient, *FLT3-ITD*: four patients, *WT1*: six patients, Monosomy 7: three patients, *NPM1*: one patient, *RAS*: one patient, *TP53*: one patient, *RUNX1-RUNX1T1*: one patient, Inv (3): one patient, *CEBPA*: two patients, *IDH1/2*: one patient, and *NUP98*: one patient) and showed no response to venetoclax regimens [103]. Finally, two AYAs, an 18-year-old male and a 20-year-old female, with *KMT2A* and *JAK1* mutations and *NPM1*, *BCORL1*, *PTPN11*, and *WT1* mutations, respectively, showed non-evaluable responses [103]. Treatment-related toxicity included febrile neutropenia (16 patients) or neutropenia (1 patient) in 17 AYA patients, sepsis in 4 AYA patients, tumor lysis syndrome and thrombocytopenia in the 15-year-old male, and pancytopenia in the 16-year-old female [103]. Mild toxicity (elevated liver enzymes: one patient, and nausea: one patient) was reported for two AYA patients, while six AYAs in this study did not display treatment-related toxicity [103].

A recently published large retrospective cohort study of children and AYAs with multiply-R/R acute leukemias treated with a venetoclax/azacitidine combination was conducted by Niswander et al., offering single-center evidence of off-label venetoclax use in this age group [104]. Venetoclax was given orally once daily at dosages of 200 mg (day 1), 400 mg (day 2), and 800 mg (days 3–28) and azacitidine at 100 mg/m^2^ daily intravenously on days 1–5 of each 28 day cycle for a total of six cycles [104]. Subsequent cycles utilized venetoclax at a full dose of 800 mg for 28 days without dose escalation [104]. Of the 37 patients treated in the fields of this cohort study, 6 were AYAs with AML [104]. Five of them, specifically a 17-year-old male with DNMT3A and GATA2 mutations, a 16-year-old male with NRAS-mutated AML, a 15-year-old male carrying a SET: NUP214 fusion gene, an 18-year-old female with a germline TP53 mutation, and an 18-year-old female with an RUNX-1 mutation, achieved CR, as evaluated by MRD [104]. One patient (19-year-old male), with monosomy 7 and NRAS mutations, presented with MRD [104]. Detailed data about the treatment-related toxicities for each patient were not analyzed, however, cytopenia in two patients, bacteremia in six patients, and fungal infections in two patients were reported [104].

Evidence exported from all the clinical trials and cohort studies previously analyzed is presented in Table 1.

## 5. Venetoclax in Mixed-Phenotype Acute Leukemia in AYAs

Mixed-phenotype acute leukemia (MPAL) is a rare hematologic malignancy (5% of all acute leukemias) characterized by the co-expression of myeloid and lymphoid antigens on the same blasts or by two separate subsets of neoplastic cells expressing distinct lineage characteristics [105,106]. MPAL’s prognosis is poor, mainly due to its rarity and the difficulties in understanding its pathogenetic physiology, and novel therapeutic regimens for MPAL in the AYA population have not currently been well-investigated. In the framework of establishing potential safe and effective treatment options, venetoclax regimens have been used in some cases of myeloid mixed-lineage acute leukemia (MPAL), including the SAVE study, which was previously described [101]. However, the rest of the current evidence is reported in the medical literature as case series.

Characteristically, Wu et al. reported two cases of AYA patients with MPAL [105]. The first case was a 23-year-old female patient with one population (61.64%) of phenotypically abnormal B-lineage blasts and a second population (29.44%) of aberrant myeloid cells, who received decitabine combined with venetoclax for induction therapy, relapsed, and was then treated with an ALL-based therapy and a venetoclax regimen as consolidation therapy, achieving complete remission after undergoing allogeneic peripheral blood stem cell transplantation [105]. The second case was a 24-year-old male, with a mixed myeloid-lymphoid population, who received venetoclax–azacitidine as induction therapy, idarubicin and cytarabine as consolidation therapy, and allogeneic stem cell transplantation, achieving complete remission [105]. Wang et al. presented the case of a 24-year-old male diagnosed with MPAL AML (64.3% myeloid blasts and 3.39% B-lineage blasts), who was treated with venetoclax and azacitidine as induction chemotherapy, achieving complete remission before undergoing an autologous stem cell transplantation [107]. Wu Xiaoxia et al. presented a series of six MPAL patients (five: B-cell/myeloid MPAL, one: B-cell/T-cell MPA, three: *KMT2A* abnormalities, and one: *BCR*: *ABL1* abnormalities), who were all given blinatumomab and venetoclax as induction therapy and, except two, achieved complete remission, as measured by MRD [108].

Evidence observed by investigating MPAL AYA patients’ chemosensitivity to venetoclax regimens is encouraging; patients appear to undergo a complete response in both frontline and relapsed disease [105,107,108]. Considering that MPAL is characterized by a poor prognosis in all age groups and that the AYA age group is less studied in terms of novel regimens and disease pathophysiology, it is suggested that these case series’ results need to be confirmed by future well-designed trials in MPAL AYA cohorts.

## 6. Discussion

The treatment of R/R AML in the AYA population remains a challenge for clinicians, despite the progress in the therapeutic evaluation of neoplastic hematologic diseases during recent decades [51,61]. The distinct characteristics of AYA patients combined with the molecular heterogeneity of AML create a difficult hematologic entity to approach [7,8,51,61,96]. Currently, fewer than half of AYA patients survive R/R AML [16,17,95,98]. While outcomes in patients’ OS have improved over time, this improvement has been attributed more to supportive care and disease complications’ management, rather than to the development of novel therapeutic regimens [32]. As patients’ survival has reached a plateau and the intensification of chemotherapy is not feasible due to treatment-related toxicity, the scientific community has had to introduce novel pathways in resolving this issue [19]. Standardizing relapse definitions and treatment-response criteria, investigating novel targeted agents, and encouraging pediatric and AYA (including populations with rare biological/molecular subtypes) enrollment in clinical trials gradually improves the diagnostic and therapeutic evaluation of R/R AML in AYAs [32,109]. Towards this direction, additional efforts towards genetic and biological characterization remain necessary.

In order to provide more insights into the highly complex nature of AML in AYAs, genome-wide approaches are currently used, taking into account the varied genomic landscape of the disease in this distinct population [110]. Array-based comparative genomic hybridization (array-CGH) and single-nucleotide polymorphism (SNP) arrays have identified genomic regions that differ from those in adult counterparts in terms of frequency, including aberrations in *WT1*, *NF1*, and *TET2* [110,111,112,113,114]. Non-targeted techniques, mainly next-generation sequencing, have been powerful in the therapeutic targeting of newly identified mutations [110]. As a result, the genomic landscape of AML has been recently updated for the age group of AYAs [32]. Novel tumor-specific therapeutic approaches with milder adverse effects compared to the standard chemotherapeutic treatment are currently being proposed and tested [110]. Besides identifying molecular targets, the molecular investigation of AML subtypes can also evaluate genetic alterations that are associated with enhanced or decreased responses to chemotherapy that targets apoptotic pathways (e.g., mitochondrial apoptosis), identifying mechanisms that modulate sensitivity to these chemotherapeutic options, including Bcl-2 inhibitors that are referred to herein [28].

As discussed in this review, targeting the apoptosis pathway by inhibiting Bcl-2 family proteins with venetoclax combined with established chemotherapy is a promising strategy in AML treatment in terms of improving the survival and minimizing the treatment-related toxicity of the AYA age group [23,26,32,64]. Our literature search revealed four clinical trials and four single-institution retrospective cohort studies including AML AYA populations. We extracted the AYA individuals in each study, where available (total: 44 subjects), aiming to draw conclusions about their characteristics, cytogenic/mutational profile, concomitant treatments, and treatment-related toxicity (Table 2). Unfortunately, for some of these parameters, especially for treatment-related toxicity, some studies had no reported results for each patient individually, but for the total cohort [99,100,101,104]. The obtained data show that venetoclax has been combined, for AML treatment in AYA cases, with various agents: cytarabine, azacytidine, decitabine, idarubicin, fludarabine, cladribine, selinexor, filgrastim, cedazuridine, revumenib, gemtuzumab ozogamicin, gilteritinib, sorafenib, midostaurin, Mcl-1 inhibitors, CDK inhibitors, and arsenic. Some of these (16) are established in AML treatment, while others (selinexor, revumenib, and sorafenib) are currently under investigation in ongoing clinical trials. According to real-world evidence, venetoclax regimens can be used both as an emergency therapy in the case of treatment-resistant leukemias and as a bridge therapy if it is necessary to perform a HSCT [115]. All studies in the AML AYA population included both relapsed and primary refractory cases. Reviewing the studies on R/R AML in AYAs, 1 of the reported cases had a prior HSCT, 13 cases had a post-chemotherapy HSCT, and 6 studies did not report data for each patient individually. In the ITCC-101/APAL2020D (NCT05183035) study, post-treatment HSCT would be available for the responding patients after the first two cycles, but the results have not been reported for each patient individually yet [100,116]. 

Data obtained from the studies that reported results for each participant reveal 44 cases treated with venetoclax regimens, with a median age of 18 years. The overall response rate varied between the different studies [96,98,99,100,101,102,103,104]. Considering the small number of studies and patients, it is early to draw conclusions; however, it is interesting that a high response rate across various cytogenetics, both relapsed and refractory cases, and almost all molecular subtypes of AML was reported [96,98,99,100,101,102,103,104]. Mutations with increased sensitivity to venetoclax regimens were identified herein, such as *IDH2*, *NPM1*, *RUNX1*, *FLT-3*, and *KMT2A* [96,98,99,100,101,102,103,104]. Significantly, the VENAML studied showed that one size does not fit all, as patients carrying *FLT3* mutations did not respond to therapy (in contrast with Trabal et al.’s study), possibly due to the lack of multiple inhibitors used for each patient, according to the karyotyping and mutational profile [99,103]. Chemotherapy combined with venetoclax is considered as an important addition: the chemotherapeutic inhibition of Bcl-XL, Mcl-1, and Bcl2-A1 proteins has been shown to increase sensitivity to venetoclax in resistant cells [116,117]. In Trabal et al.’s cohort, an example of CR using venetoclax combined with an Mcl-1 inhibitor was presented [103]. Even ignoring each patient’s mutational profile, Mcl-1 inhibitors’ use may be beneficial in terms of survival, considering the upregulation of different pro-survival Bcl-2 family members as a result of venetoclax use in some studies [118,119,120] and the Mcl-1 amplification and overexpression seen in pre-clinical studies [118,119,120,121,122]. Moreover, a study by Niu et al. revealed that cytarabine or daunorubicin combined with venetoclax treatment resulted in increased DNA damage and a better reduction in Mcl-1 levels in AML cell lines than during venetoclax monotherapy [121]. Finally, considering the distinctiveness of the mechanisms mediating energy metabolism at the different developmental stages of leukemic cells, it has been shown that, in monocytic AML, cells can switch from Bcl-2 to Mcl-1 dependence to drive energy metabolism as cells obtain a higher developmental state. This favors the use of Mcl-1 inhibitors as a potential strategy to defeat venetoclax resistance in monocytic AML [85]. 

It is worth mentioning that, except for Mcl-1’s involvement in venetoclax resistance, other mechanisms of resistance favor the use of potential targets as combination therapy with venetoclax, a strategy that has been shown to be more effective than venetoclax monotherapy. *RAS* mutations were shown to be associated with venetoclax resistance, as monotherapy or combined with azacitidine [85]. *TP53* mutations in leukemic stem cells disturbed mitochondrial homeostasis by impairing BAX/BAK function, decreasing venetoclax targets (Bcl-2), while *TP53* biallelic mutations were commonly described in patients who were resistant to venetoclax [79,123]. FLT3-ITD or PTPN11 mutations correlated with venetoclax resistance, possibly due to the increase in Bcl-XL and Mcl-1 protein levels [124,125,126]. Moreover, it has been shown that these mutations are acquired from AML patients during the disease relapse phase [127]. Combining FLT3 inhibitors with VTX could be a useful strategy to overcome Bcl-2 inhibitor resistance in FLT3-mutated AML patients and to prevent the appearance of FLT3-mutated subclones in patients with R/R AML [96,98,99,100,101,102,103,104]. All these mutations were described in the AYA patients of the cohorts reported herein, for whom, however, survival differed, partly due to the use of different venetoclax combinations in each study [96,98,99,100,101,102,103,104]. Unfortunately, mutations associated with venetoclax sensitivity in older adults are not frequent in the AYA population, which makes it important to determine markers of venetoclax regimen response by conducting further studies in larger AYA cohorts [10,11].

Regarding toxicity to venetoclax regimens, the most common adverse effects of the regimens used in the different studies were febrile neutropenia (17/44 patients), followed by sepsis (4/44 patients), neutropenia (3/44 patients), thrombocytopenia, pancytopenia, and tumor lysis syndrome (1/44 patients each), and milder adverse reaction in some cases (nausea and elevated liver enzymes: 1/44 patients each) [96,98,99,100,101,102,103,104]. No difference in toxicity was noted between relapsed patients and patients with primary refractory AML [96,99,100,101]. The primary toxicity of venetoclax regimens (febrile neutropenia) raises the concern of the potential risk of infections after HSCT, especially invasive fungal infections [98]. This complicates the routine use of antifungal prophylaxis in patients who are on venetoclax regimens. Venetoclax is a CYP3A4 substrate, potentiating the risk of interactions with antifungal drugs which are strong CYP3A4 inhibitors [98]. In Trabal et al.’s cohort, a venetoclax dose modification regarding antifungal treatment use was applied [103]. Dose modification is usually proposed for patients who receive concomitant antifungal treatment, however, there are no currently proposed means for monitoring venetoclax serum levels in clinical practice [98]. 

## 7. Conclusions

In this review, we gathered the data of the last two decades published on the use of venetoclax in the special population of AYAs (summarized in Table 1 and Table 2). Venetoclax combination therapy is an optimal chemotherapeutic regimen for R/R AML in AYAs, considering its positive effect. The available real-world data concerning its efficacy and safety are encouraging. However, high-quality evidence for venetoclax’s incorporation into second-line treatment is still lacking, and the role of the relevant genetic differences between AYAs and the pediatric and adult age groups in its efficacy still needs further investigation. Formal clinical trial research, including optimal dosing, treatment duration, and pharmacokinetic analysis. is needed, in order to demonstrate the benefit of venetoclax use in this chemorefractory distinct population. Finally, a determined focus on evaluating the underpinnings of molecular genetics and epigenetics in the AYAs group could potentially encourage their participation in clinical trials, offering the maximum beneficial outcomes and providing further gains in survival.

## Figures and Tables

**Table 1:** Clinical trials (recently completed or ongoing) and retrospective cohort studies for pediatric, adolescents and young adult populations with an AML diagnosis treated with venetoclax regimens. For the purposes of this review only data for AYA patients were extracted and reported herein. Patients’ characteristics (age/sex), cytogenics and molecular characteristics, venetoclax dosage and concomitant medication, HSCT status, response, and treatment related toxicity for each patient (when available) are presented diagrammatically. (CR: Complete Response/CRi: Complete Response without blood count recovery, F: Female, M: Male, SCT: Stem Cell Transplantation, NR: Non-Response, PR: Partial Response).

Study	Phase and Population	AYAs Number (N)	Patient Serial Number for the Purpose of This Review	Age/Sex	Cytogenics and Molecular Characteristics	Dose	Concomitant Medication	Prior/Post Treatment SCT	Efficacy	Toxicity
VENAML (NCT03194932) [99]	I/II	6	1	18/F	FUS-ERG	240 mg/m^2^ or 360 mg/m^2^ Phase 2: 360 mg/m^2^ (maximum: 600 mg/m^2^)	Cytarabine, with or without Idarubicin	−	CR	Not analyzed for each patient, febrile neutropenia in 63% of the total 38 pediatric and AYAs participants
VENAML (NCT03194932) [99]	I/II	6	2	17/M	FLT3-ITD, MECOM, CBL, PTPN11, WT1	240 mg/m^2^ or 360 mg/m^2^ Phase 2: 360 mg/m^2^ (maximum: 600 mg/m^2^)	Cytarabine, with or without Idarubicin	−	NR	Not analyzed for each patient, febrile neutropenia in 63% of the total 38 pediatric and AYAs participants
VENAML (NCT03194932) [99]	I/II	6	3	15/F	dup(2)(q11.2q21); CEBPA, IKZF1	240 mg/m^2^ or 360 mg/m^2^ Phase 2: 360 mg/m^2^ (maximum: 600 mg/m^2^)	Cytarabine, with or without Idarubicin	−	PR	Not analyzed for each patient, febrile neutropenia in 63% of the total 38 pediatric and AYAs participants
VENAML (NCT03194932) [99]	I/II	6	4	16/M	PICALM-MLLT10; TP53, EXH2, PTPN11, NF1, PHF6	240 mg/m^2^ or 360 mg/m^2^ Phase 2: 360 mg/m^2^ (maximum: 600 mg/m^2^)	Cytarabine, with or without Idarubicin	−	PR	Not analyzed for each patient, febrile neutropenia in 63% of the total 38 pediatric and AYAs participants
VENAML (NCT03194932) [99]	I/II	6	5	19/M	KMT2A	240 mg/m^2^ or 360 mg/m^2^ Phase 2: 360 mg/m^2^ (maximum: 600 mg/m^2^)	Cytarabine, with or without Idarubicin	+ (prior treatment)	CR	Not analyzed for each patient, febrile neutropenia in 63% of the total 38 pediatric and AYAs participants
VENAML (NCT03194932) [99]	I/II	6	6	17/F	RUNX1-RUNX1T1	240 mg/m^2^ or 360 mg/m^2^ Phase 2: 360 mg/m^2^ (maximum: 600 mg/m^2^)	Cytarabine, with or without Idarubicin	−	CR	Not analyzed for each patient, febrile neutropenia in 63% of the total 38 pediatric and AYAs participants
SELCLAX (NCT04898894) [96]	I	2	1	17/F	NOP98, NSD1, FLT3-ITP	360 mg/m^2^ (max 600 mg)	Selinexor	−	PR	No
SELCLAX (NCT04898894) [96]	I	2	2	16/F	KMT2A, MLT3	360 mg/m^2^ (max 600 mg)	Selinexor	−	NR	No
ITCC-101/APAL2020D (NCT05183035) [100]	III	Not #reported	N/A	N/A	Absence of FLT3/ITD mutation	C1D1: 300 mg, 600 mg on subsequent days of each 28 day cycle	Fludarabine/ Cytarabine, Filgrastim	+ (post treatment: for the responding patients after the first 2 cycles)	Not reported	Not reported
SAVE (NCT05360160) [101]	I/II	Not reported	N/A	Median age: 27	Not reported	400 mg daily	Decitabine/ Cedazuridine, Revumenib	+ (post treatment)	Not reported	Not reported
Winters et al. [102]	Retrospective cohort study	2	1	18/F	FLT3-ITD, WT1, NUP98-NSD1	Day 1: 100 mg, Day 2: 200 mg, Day 3: 400 mg, and 400 mg for 28-days cycles	Azacitidine	+ (post treatment)	CR	Neutropenia
Winters et al. [102]	Retrospective cohort study	2	2	20/M	FLT3-TKD, WT1, BCORL1, GATA2, 3 46, XY, t (2;14) (q22; q32)	Day 1: 100 mg, Day 2: 200 mg, Day 3: 400 mg, and 400 mg for 28-days cycles	Azacitidine	+ (post treatment)	CR	Neutropenia
Báez-Gutiérrez [98]	Retrospective cohort study	1	1	26/F	t (6; 11), mixed-lineage leukaemia (MLL) positive, FLT3-ITD negative	Up titrated from 100 mg daily to a maximum dose of 400 mg (3 cycles)	Azacitidine	+ (post treatment)	NR	Febrile syndrome
Trabal et al. [103]	Retrospective cohort study	27	1	15/M	FLT3-ITD, NUP98-NSD1, PTPN11, WT1	63–138 mg/m^2^ (6 cycles)	Azacitidine, Cladribine, Idarubicin, Gemtuzumab ozogamicin, CDK inhibitor	−	NR	Tumor lysis syndrome, thrombocytopenia, febrile neutropenia
Trabal et al. [103]	Retrospective cohort study	27	2	16/F	KMT2A, STAT5	75 mg/m^2^ (2 cycles)	Azacitidine	−	PR	Pancytopenia, Sepsis
Trabal et al. [103]	Retrospective cohort study	27	3	17/F	t(9;11), KMT2A, PRPF40B, WT1	153 mg/m^2^ (1 cycle)	Azacitidine, Cladribine, Idarubicin, Gemtuzumab ozogamicin	+ (post treatment)	CRi	Febrile neutropenia
Trabal et al. [103]	Retrospective cohort study	27	4	17/M	FLT3-ITD, inversion 3, monosomy 7, CALR, CBL, PTPN11, STAT5#A, WT1	62–124 mg/m^2^ (2 cycles)	Fludarabine, Cytarabine, Granulocyte-colony stimulating factor, Gemtuzumab ozogamicin Tyrosine kinase inhibitor, Cladribine, Cytarabine, Arsenic	−	NR	Febrile neutropenia
Trabal et al. [103]	Retrospective cohort study	27	5	17/M	KMT2A, PTPN11	56 mg/m^2^ (4 cycles)	Azacitidine	−	NR	No
Trabal et al. [103]	Retrospective cohort study	27	6	17/F	None	263 mg/m^2^ (1 cycle)	Azacitidine	−	NR	Neutropenia
Trabal et al. [103]	Retrospective cohort study	27	7	18/M	IKZF1, NF1, PTPN11, DNMT3A	122 mg/m^2^ (3 cycles)	Decitabine	−	NR	Febrile neutropenia
Trabal et al. [103]	Retrospective cohort study	27	8	18/M	KMT2A	67 mg/m^2^ (1 cycle)	Decitabine	−	NR	No
Trabal et al. [103]	Retrospective cohort study	27	9	18/M	KMT2A, JAK1	167 mg/m^2^ (1 cycle)	Azacitidine	+ (post treatment)	NE	No
Trabal et al. [103]	Retrospective cohort study	27	10	18/F	MECOM(EVI1), Inv 3, monosomy 7, CUX1, WT1, PTNP11	41–117 mg/m^2^ (2 cycles)	Fludarabine, Cytarabine, Granulocyte-colony stimulating factor, Idarubicin, Mcl-1 inhibitor	−	NR	Elevated liver enzymes
Trabal et al. [103]	Retrospective cohort study	27	11	19/M	MECOM(EVI1)r, Inv 3, monosomy 7, NRAS, WT1	46–92 mg/m^2^ (3 cycles)	Decitabine	+ (post treatment)	CRi	Febrile neutropenia
Trabal et al. [103]	Retrospective cohort study	27	12	19/M	t (3;3), monosomy 7, FLT3-ITD, BCORL1, PTPN11	126 mg/m^2^ (16 cycles)	Fludarabine, Cytarabine, Granulocyte-colony stimulating factor, Tyrosine kinase inhibitor, Decitabine	−	NR	Febrile neutropenia, nausea
Trabal et al. [103]	Retrospective cohort study	27	13	20/F	NPM1, t(4;8), t(7;8), BCORL1, PTPN11, WT1	118 mg/m^2^ (1 cycle)	Decitabine	−	NE	No
Trabal et al. [103]	Retrospective cohort study	27	14	20/F	WT1	90 mg/m^2^ (2 cycles)	Decitabine	−	NR	No
Trabal et al. [103]	Retrospective cohort study	27	15	20/F	NRAS, KRAS	50 mg/m^2^ (4 cycles)	Azacitidine	+ (post treatment)	CRi	Febrile neutropenia
Trabal et al. [103]	Retrospective cohort study	27	16	20/M	Monosomy 7, KRAS	47–93 mg/m^2^ (10 cycles)	Decitabine, Azacitidine, Cladribine, Idarubicin, Gemtuzumab ozogamicin	+ (post treatment)	CRi	Febrile neutropenia
Trabal et al. [103]	Retrospective cohort study	27	17	20/M	KMT2A, SMC1A	110 mg/m^2^ (2 cycles)	Fludarabine, Cytarabine, Granulocyte-colony stimulating factor, Idarubicin	+ (post treatment)	CRi	Febrile neutropenia
Trabal et al. [103]	Retrospective cohort study	27	18	20/M	FLT3-ITD, IDH2; NPM1	105 mg/m^2^ (1 cycle)	Decitabine, Gemtuzumab ozogamicin	−	NR	No
Trabal et al. [103]	Retrospective cohort study	27	19	20/F	KMT2A	75 mg/m^2^ (2 cycles)	Azacitidine	−	CR	Sepsis
Trabal et al. [103]	Retrospective cohort study	27	20	21/M	IDH2	44 mg/m^2^ (2 cycles)	Fludarabine, Cytarabine, Granulocyte-colony stimulating factor, Idarubicin	+ (post treatment)	CR	Febrile neutropenia, sepsis
Trabal et al. [103]	Retrospective cohort study	27	21	21/F	FLT3-1868a, PIGA, WT1	60 mg/m^2^ (1 cycle)	Azacitidine, Gilteritinib	−	NR	Febrile neutropenia
Trabal et al. [103]	Retrospective cohort study	27	22	21/F	RUNX1-RUNX1T1;	118 mg/m^2^ (4 cycles)	Decitabine, Gemtuzumab ozogamicin	−	CR	Febrile neutropenia
Trabal et al. [103]	Retrospective cohort study	27	23	21/M	RUNX1-RUNX1T1, CEBPA, KIT, STATSA	60 mg/m^2^ (1 cycle)	Decitabine	−	NR	Sepsis
Trabal et al. [103]	Retrospective cohort study	27	24	21/F	FLT3-ITD, NPM1, RUNX1, SH2B3, TP53, WT1	134–268 mg/m^2^ (4 cycles)	Decitabine, Tyrosine kinase inhibitor, Azacitidine	+ (post treatment)	CR	Febrile neutropenia
Trabal et al. [103]	Retrospective cohort study	27	25	21/M	CEBPA, WT1	37 mg/m^2^ (2 cycles)	Cytarabine, Decitabine	−	NR	Febrile neutropenia
Trabal et al. [103]	Retrospective cohort study	27	26	21/M	KRAS, NRAS, BRINP3, TP53	52 mg/m^2^ (1 cycle)	Decitabine	−	NR	Febrile neutropenia
Trabal et al. [103]	Retrospective cohort study	27	27	21/M	NPM1, GATA2	50 mg/m^2^ (2 cycles)	Azacitidine, Cladribine, Idarubicin	+ (post treatment)	CRi	Febrile neutropenia
Niswander et al. [104]	Retrospective cohort study	6	1	17/M	DNMT3A, GATA2	Day 1: 200 mg Day 2: 400 mg Days 3–28: 800 mg, 6 cycles	Azacitidine	Not reported	CR	Not analyzed for each patient, for the total of the 37 patients: cytopenia (2 patients), bacteremia (6 patients) and fungal infections (2 patients) were reported
Niswander et al. [104]	Retrospective cohort study	6	2	16/M	NRAS	Day 1: 200 mg Day 2: 400 mg Days 3–28: 800 mg, 6 cycles	Azacitidine	Not reported	CR	Not analyzed for each patient, for the total of the 37 patients: cytopenia (2 patients), bacteremia (6 patients) and fungal infections (2 patients) were reported
Niswander et al. [104]	Retrospective cohort study	6	3	15/M	SET: NUP214 fusion	Day 1: 200 mg Day 2: 400 mg Days 3–28: 800 mg, 6 cycles	Azacitidine	Not reported	CR	Not analyzed for each patient, for the total of the 37 patients: cytopenia (2 patients), bacteremia (6 patients) and fungal infections (2 patients) were reported
Niswander et al. [104]	Retrospective cohort study	6	4	18/F	TP53	Day 1: 200 mg Day 2: 400 mg Days 3–28: 800 mg, 6 cycles	Azacitidine	Not reported	CR	Not analyzed for each patient, for the total of the 37 patients: cytopenia (2 patients), bacteremia (6 patients) and fungal infections (2 patients) were reported
Niswander et al. [104]	Retrospective cohort study	6	5	18/F	RUNX-1	Day 1: 200 mg Day 2: 400 mg Days 3–28: 800 mg, 6 cycles	Azacitidine	Not reported	NR	Not analyzed for each patient, for the total of the 37 patients: cytopenia (2 patients), bacteremia (6 patients) and fungal infections (2 patients) were reported
Niswander et al. [104]	Retrospective cohort study	6	6	19/M	Monosomy 7, NRAS	Day 1: 200 mg Day 2: 400 mg Days 3–28: 800 mg, 6 cycles	Azacitidine	Not reported	NR	Not analyzed for each patient, for the total of the 37 patients: cytopenia (2 patients), bacteremia (6 patients) and fungal infections (2 patients) were reported

**Table 2:** Characteristics of studies, patients, and chemotherapeutic regimens. This quantitative analysis was performed excluding the studies that did not refer the total number of AYA patients. [* In ITCC-101/APAL2020D (NCT05183035) study, post treatment HSCT will be available for the responding patients after the first 2 cycles (pending results, the primary analysis will be performed approximately 5 years after the first patient is randomized), ** In ITCC-101/APAL2020D (NCT05183035) and SAVE (NCT05360160) studies results were not yet reported for each patient individually, *** Toxicity in studies is not reported for each patient individually. We report results observed for the total cohort underlying neutropenia as the most common adverse reaction to venetoclax regimens].

Studies’, Patients’, and Regimens’ Characteristics	Number of Patients Reported
Number of studies	8
Clinical trials	4
Completed	1
Ongoing	3
Cohort studies	4
Single-institution	3
Multi-center	1
Number of concomitant drugs	19
On-label	16
Off-label	3
Number of subjects reported	44
HSCT	14
Prior-treatment	1
Post-Treatment *	13
Not reported	6
Median age	18
High risk cytogenics	
Overall response **	
Complete response	19
Partial response	4
Non-response	19
Not evaluable response	2
Toxicity ***	24
Febrile neutropenia	17
Neutropenia	3
Sepsis	4
Thrombocytopenia	1
Pancytopenia	1
Tumor lysis syndrome	1
Nausea	1
Elevated liver enzymes	1
